# Development and external validation of a novel prediction model for the TraumaTriage App

**DOI:** 10.1007/s00068-026-03175-8

**Published:** 2026-04-17

**Authors:** Max Gulickx, Robin D. Lokerman, Rogier van der Sluijs, Rinske M. Tuinema, Risco van Vliet, Falco Hietbrink, Rolf H. H. Groenwold, Mark van Heijl

**Affiliations:** 1 Department of Surgery, University Medical Center Utrecht, C04.332, Heidelberglaan 100, Utrecht, 3584 CX The Netherlands; 2 Department of Radiology, University Medical Center Utrecht, Utrecht, The Netherlands; 3 Regional Ambulance Facilities Utrecht, Bilthoven, The Netherlands; 4 Department of Emergency Medicine, Diakonessenhuis Utrecht/Zeist/Doorn, Utrecht, The Netherlands; 5 Regional Ambulance Facilities Brabant Midden-West-Noord, ’s- Hertogenbosch, The Netherlands; 6 Trauma Center Utrecht, Utrecht, The Netherlands; 7 Department of Clinical Epidemiology, Leiden University Medical Center, Leiden, The Netherlands; 8 Department of Surgery, Diakonessenhuis Utrecht/Zeist/Doorn, Utrecht, The Netherlands

**Keywords:** Trauma triage, Prediction model, Pre-hospital care, Machine learning, Severe injury

## Abstract

**Purpose:**

Accurate pre-hospital trauma triage is essential for optimizing survival and functional outcomes within inclusive trauma systems. A prediction model incorporated into the TraumaTriage App (TTApp) has been shown to improve patient allocation in the Netherlands, but was developed based on a relatively small cohort. This study aims to redevelop and improve the performance and applicability of the TTApp’s prediction model before nationwide implementation.

**Methods:**

In this prospective multicenter cohort study, data from all trauma patients transported within two emergency medical service (EMS) regions (i.e., Brabant and Utrecht), between February 2015 and October 2019, were used to develop and externally validate a prediction model to identify severely injured adults (Injury Severity Score [ISS] ≥ 16) using routinely available pre-hospital data. A Gradient Boosting Decision Tree (GBDT) algorithm was applied, and model performance was evaluated in terms of discrimination and calibration.

**Results:**

The development cohort included 51,001 patients (median age 63.8 years, median ISS 9), and the external validation cohort included 29,737 patients (median age 62.1 years, median ISS 9). In external validation, the GBDT model showed excellent discrimination (c-statistic 0.850; 95% CI, 0.837–0.863) and good calibration (calibration-in-the-large 0.009; slope 0.952). Sensitivity was 91.3% and 85.2% at specificity thresholds of 50% and 65%, respectively.

**Conclusions:**

This externally validated prediction model effectively identifies severely injured patients in the pre-hospital setting and represents a first iteration towards enhancing the TTApp. Designed for use in urgent situations, the model allows predictions with incomplete data and shows potential to reduce undertriage rates toward 10% while maintaining acceptable overtriage levels.

**Supplementary Information:**

The online version contains supplementary material available at 10.1007/s00068-026-03175-8.

## Background

Accurate pre-hospital triage of trauma patients is crucial for patient outcomes, as the transportation of a severely injured patient to a lower-level trauma center (i.e., undertriage) is associated with increased mortality and lifelong disabilities [1]. Conversely, the transportation of mildly or moderately injured patients to a higher-level trauma center (i.e., overtriage) results in increased costs and overutilization of scarce resources [2]. The Dutch Health Care Institute and the American College of Surgeons Committee on Trauma (ACSCOT) therefore set a maximum undertriage rate of 10% and even 5%, respectively [3, 4]. Unfortunately, no inclusive trauma system has been able to adhere to these standards [5].

Pre-hospital triage is – in most inclusive trauma systems – performed by Emergency Medical Service (EMS) professionals who assess a patient’s need for specialized trauma care at the scene of injury and subsequently determines transportation destination. In the Netherlands, EMS professionals are aided in their on-scene decision making by the field triage criteria of the Dutch National Protocol of Ambulance Services [6], which is derived from the American Field Triage Decision Scheme [4, 7]. These decision schemes, however, provide limited assistance as they showed to be insensitive (i.e., sensitivity 36.2%, specificity 92.6%) in identifying severely injured patients [8].

We previously developed an algorithm that was potentially able to decrease undertriage to approximately 10%, while maintaining an overtriage rate of 50% [9]. When integrated in a mobile application (Trauma Triage App [TTApp]) and implemented in the Dutch pre-hospital practice, the use of the TTApp demonstrated to decrease undertriage rates with 5% from 32% to 27%, while maintaining overtriage at approximately 20% [10]. However, the performance of the TTApp’s prediction model could, potentially, be further improved using machine learning and a larger study population as part of a continuous improvement strategy, as the previous model was developed using a relatively small sample size and conventional modeling technique.

In the current study, we aim to redevelop and externally validate a prediction model to identify severely injured patients to improve the performance and applicability of the TTApp before nationwide implementation.

## Methods

### Study setting

This prospective, multicenter cohort study - performed between February 1, 2015, and October 31, 2019 - was conducted during the implementation study of the TTApp in two EMS regions (Brabant Midden-West-Noord and Utrecht). The corresponding ambulance services fully cover 3 of the 11 inclusive trauma regions in the Netherlands (Traumazorgnetwerk Midden-Nederland, Netwerk Acute Zorg Brabant, and Acute Zorgregio Oost) non-exclusively [10]. The participating trauma regions comprise 3 higher-level (i.e., level-1) and 18 lower-level (i.e., level 2 or 3) trauma centers. The ambulance services transport around 160,000 trauma patients to a trauma center annually and serve a region of rural, suburban, and urban surroundings in an area of approximately 5000 km2 that accommodates around 4 million people [11].

### Patients

All trauma patients aged 16 years or older who were transported by the participating ambulance services to a trauma center in the participating trauma regions, were included. Patients transported to a trauma center in a non-participating trauma region were excluded as data were unavailable, with the transportation to these regions most likely being related to geographical factors. Patients transported by ambulance service Brabant Midden-West-Noord were included between May 1, 2016, and October 31, 2019, and patients transported by ambulance service Utrecht were included between February 1, 2015, and September 30, 2019.

### Outcomes

The primary objective was to develop a prediction model based on ISS ≥ 16, a universally recognized threshold to define severe injury and the criterion recommended by the ACSCOT for pre-hospital triage evaluation [4]. Undertriage is defined as a severely injured patient (ISS ≥ 16) transported to a lower-level trauma center, whereas overtriage is defined as a non-severely injured patient (ISS < 16) transported to a higher-level trauma center.

### Data collection

Pre-hospital and hospital data were prospectively collected during the implementation of the TTApp in the TraumaTriage Intervention study [10], and were linked using a previously developed linkage tool with an externally validated accuracy of 100% (95%-CI; 100.0–100.0) [12]. Pre-hospital data contained patients’ demographics, vital parameters, description of the injury mechanism, transportation destination, and a free text in which the physical examination and suspected injuries were described. Trained research assistants read all free texts and classified, independent of each other, whether the EMS professional suspected a serious injury (AIS ≥ 2) in the head or neck, thorax, abdomen, pelvic, or extremity region. Hospital data of the three participating trauma regions were prospectively collected by the Dutch Trauma Registry and comprised – among others – all diagnosed injuries within 30-days after trauma, and mortality. Professional data managers of the trauma registries classified injuries using the Abbreviated Injury Score (AIS) and computed ISSs [13].

### Predictors

Predictors associated with severe injury were chosen based on clinical reasoning and previous research [9, 10]. The maximum number of predictors was limited to ensure practical applicability when incorporated in the TTApp. The prediction model was developed based on the following 12 predictors within (1) patient demographics (i.e., age), (2) vital signs (i.e., systolic blood pressure, oxygen saturation, and Glasgow Coma Scale pre-sedation and intubation), (3) injury mechanism (i.e., high-energy mechanism criteria), (4) the pre-hospital suspicion of serious injuries (i.e., suspicion of serious head or neck injury, thoracic injury, abdominal injury, pelvic fracture, extremity injury, or suspected serious injury in > 1 region), and (5) ambulance dispatch priority (Table 1).

**Table 1:** Predictor variables

**Demographics**
Age
**Vital Signs**
Systolic blood pressure
Oxygen Saturation
Glasgow Coma Scale
**Injury mechanism**
Mechanism criteria*
**Suspected serious injury in AIS region**
Head/neck region
Thorax region
Abdomen region
Pelvic region
Extremities region
Multiple regions (> 1 region)
**Urgency**
Ambulance dispatch priority

^*^ Mechanism criteria include high-energy trauma mechanism such as a fall > 2-meter, motor vehicle crash > 32 km/h, or any type of entrapment

### Missing data

Missing data are inherent and unavoidable in pre-hospital triage settings due to the urgent nature of providing trauma care. This often leads to challenges in the practical application of prediction models relying on logistic regression, as these models require complete data input to generate predictions. A GBDT algorithm was therefore used in the current study, as it deals with missing data by default by selecting the split-direction of a node that minimizes training loss (i.e., sparsity-aware split finding), consequently allowing the model to predict the presence of severe injury when certain parameters (e.g., vital signs) are not available [14].

### Statistical analysis

R statistical software (version 4.0.3.) was used to perform all statistical analyses [15]. The XGBoost package was used to develop a GBDT model to predict the presence of severe injury (ISS ≥ 16). A GBDT model is a type of machine learning algorithm which generally provide highly accurate predictions, as it works by combining multiple decision trees in which each subsequential tree is trained with the residual errors of the previous tree, improving its accuracy and creating a robust prediction model (Appendix 1) [14]. The model was trained in the Brabant region and externally validated in the Utrecht region.

The performance of the final model was estimated and described in terms of discrimination and calibration. Discrimination was determined using the concordance-statistic (c-statistic) and – after plotting the receiver operating characteristic curve (ROC-curve) –undertriage rates (i.e., 1 – sensitivity) were assessed using predefined values for acceptable overtriage rates of 50% and 35% (i.e., 1 – specificity). Calibration was determined in terms of the calibration-in-the-large and calibration slope and was depicted in a calibration plot (Fig. 2). Calibration is essential when externally validating a prediction model as it refers to the agreement between the predicted probabilities and observed outcomes, ensuring that the model’s predicted probabilities accurately resemble to the true risk of severe injury in the population it is applied to. A decision curve analysis was performed to assess net benefit across a range of threshold probabilities in the development and validation cohorts [16].

## Results

### Development of triage algorithm

**Fig. 1:**
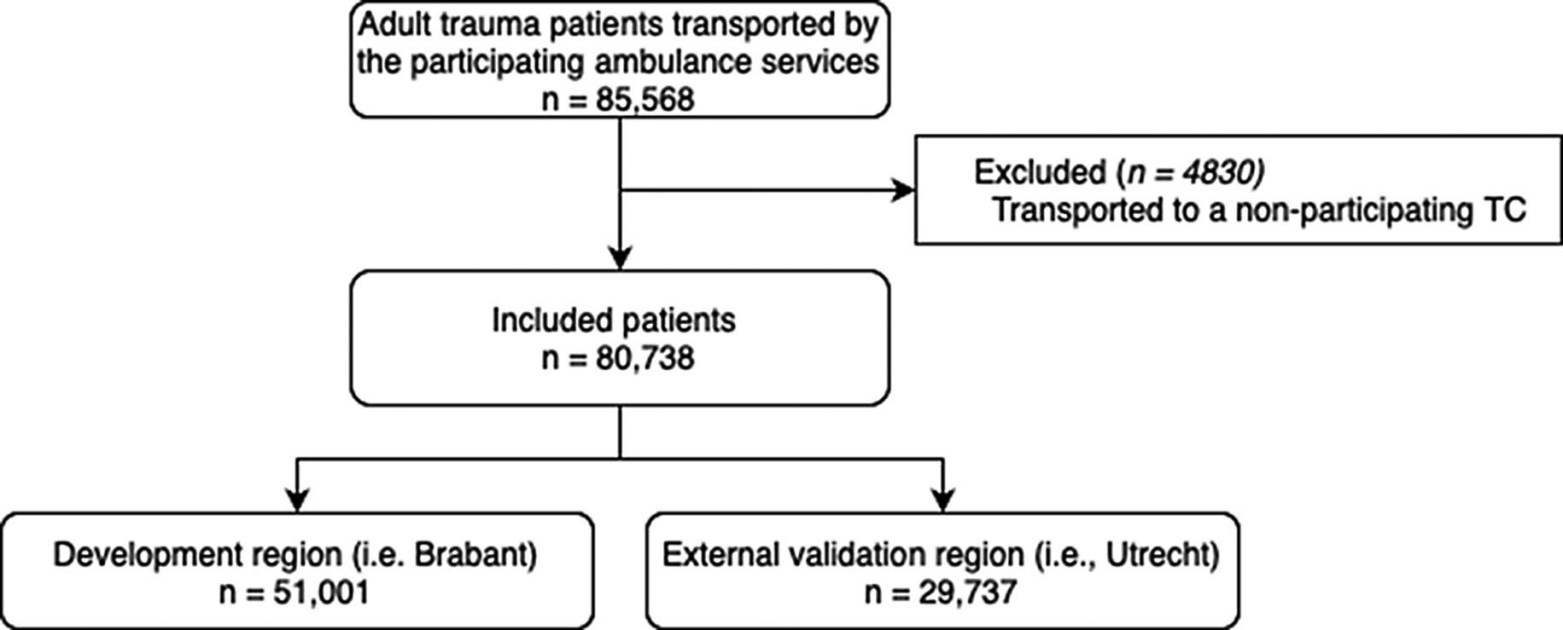
Flowchart of patient inclusion in the development and validation study of Trauma Triage Prediction Model

A total of 51,001 patients were included in the Brabant region during the study period, comprising the development data set (Fig. 1). The median age of these patients was 63.8 years (IQR; 41.0–79.9), 25,949 (50.9%) patients were male, and the median ISS was 9 (IQR; 4–9). A total of 1238 (2.4%) of the patients were severely injured according to the primary outcome (ISS ≥ 16), and undertriage and overtriage rates were 29.4% and 24.9%, respectively (Table 2). Model performance is shown in Table 3. Development of the prediction model showed a c-statistic of 0.884 (95% CI, 0.874–0.894), with sensitivity of 95.0% and 89.5% at specificity cut-off thresholds of 50% and 65%, respectively.

**Table 2:** Baseline characteristics of adult trauma patients who were transported by participating EMS to an Emergency Department

Variables	Brabant region (development)	Utrecht region (validation)
	*n* = 51,001	*n* = 29,737
**Demographics**	**Median (IQR)**	**Median (IQR)**
Age, y (median, IQR)	63.8 (41.0–79.9)	62.1 (38.1–80.0)
> 65 (n, %)	24,741 (48.5)	13,802 (46.4)
Man, (n, %)(	25,949 (50.9)	14,169 (47.7)
ISS (median, IQR)	9 (4–9)	9 (4–10)
**Pre-hospital vital parameters**	**Median (IQR)**	**Median (IQR)**
Systolic blood pressure, mmHg	141 (126–160)	142 (127–161)
Heart Rate	81 (72–93)	80 (71–90)
Respiratory rate	16 (14–18)	16 (14–18)
Glasgow Coma Scale	15 (15–15)	15 (15–15)
	**N (%)**	**N (%)**
Systolic blood pressure < 90mmHg	538 (1.1)	405 (1.4)
Heart rate > 110/min	3185 (6.2)	1580 (5.3)
Respiratory rate < 10 or > 29/min	724 (1.4)	432 (1.5)
Glasgow Coma Scale < 13	1708 (3.4)	1176 (4.0)
Revised Trauma Score < 12	2247 (4.4)	1602 (5.4)
**Mechanism of injury**	**N (%)**	**N (%)**
Mechanism criteria ^a^	1216 (2.5)	1554 (5.2)
Penetrating	289 (0.6)	263 (0.9)
Burns or inhalation injury	105 (0.2)	254 (0.9)
**Suspected serious injury in AIS region**	**N (%)**	**N (%)**
Head or Neck	6160 (12.1)	5700 (19.2)
Face	976 (1.9)	2039 (6.9)
Thorax	1985 (3.9)	2011 (6.8)
Abdomen	616 (1.2)	639 (2.1)
Pelvic	564 (1.1)	319 (1.1)
Extremities	11,361 (22.3)	12,835 (43.2)
Multiple regions	2475 (4.9)	2668 (9.0)
HEMS assistance	1062 (2.1)	225 (0.8)
**Clinical characteristics**	**N (%)**	**N (%)**
ISS ≥ 16	1238 (2.4)	920 (3.1)
Undertriage ^b^	365 (29.4)	272 (29.6)
Overtriage ^c^	12,379 (24.9)	3862 (13.4)
Transportation Destination		
Higher-level trauma center	13,246 (26.0)	4510 (15.2)
Lower-level trauma center	37,728 (74.0)	25,227 (84.8)
Highest dispatch priority	19,838 (38.9)	10,018 (33.7)
Distance closest higher-level trauma center	31.4 (21.3–42.8)	17.6 (9.9–24.4)
Pre-hospital intubation	380 (0.7)	327 (1.1)
Emergency Intervention	193 (0.4)	150 (0.5)
Admission to Intensive Care	1358 (2.7)	1107 (3.7)
24 h mortality	83 (0.2)	76 (0.3)

Abbreviations: ISS, Injury Severity Score

^a^ Mechanism criteria include a fall > 2-meter, motor vehicle crash > 32 km/h, or any type of entrapment

^b^ Severely injured patient (ISS ≥ 16) transported to a lower-level trauma center

^c^ Non-severely injured patient (ISS < 16) transported to a higher-level trauma center

**Table 3:** Performance of Trauma Triage Prediction Model at development and external validation

Performance	Development	External validation
**Discrimination**		
C-statistic, 95%-CI	0.884 (0.874–0.894)	0.850 (0.837–0.863)
Sensitivity		
Sensitivity, at 50% Specificity*	95.0%	91.3%
Sensitivity, at 65% Specificity*	89.5%	85.2%
Undertriage		
Undertriage, at 50% Overtriage	5.0%	8.7%
Undertriage, at 35% Overtriage	10.5%	14.8%
**Calibration**		
Calibration-in-the-large		0.009
Calibration Slope		0.952
**Overall performance**		
Brier score		0.025

*Sensitivity at cut-off specificity

### External validation of triage algorithm

**Fig. 2:**
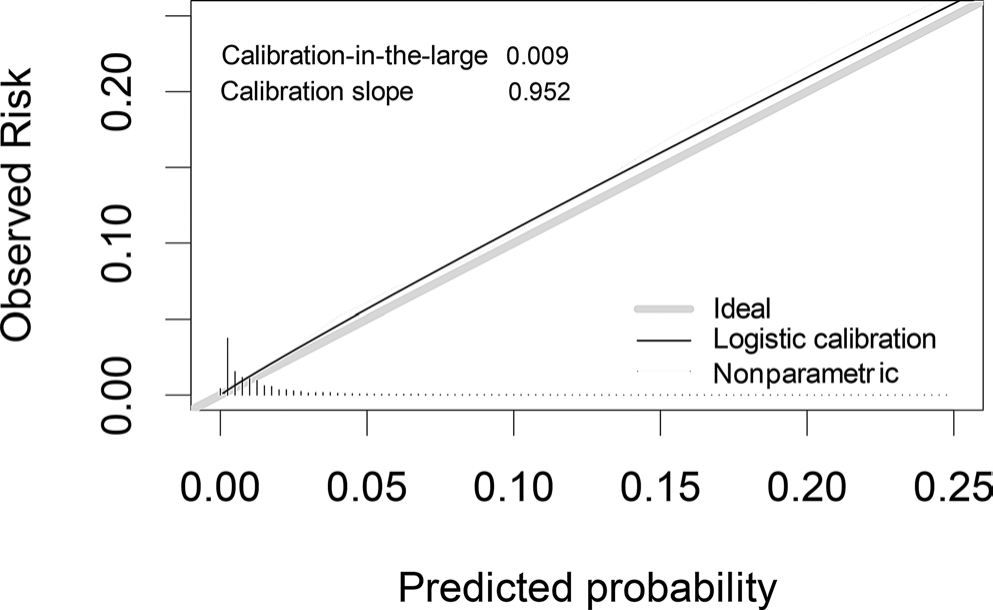
Calibration plot of the prediction model at external validation (using data from the Utrecht region)

A total of 29,737 patients were included in the Utrecht region during the study period, comprising the validation data set. The median age of these patients was 62.1 (IQR; 38.1–80.0), 14,169 (47.7%) patients were male, and the median ISS was 9 (IQR: 4–10). A total of 920 (3.1%) patients were severely injured according to the primary outcome (ISS ≥ 16), and undertriage and overtriage rates were 29.6% and 13.4%, respectively. External validation of the prediction model showed a c-statistic of 0.850 (95% CI, 0.837–0.863) and sensitivity of 91.3% and 85.2%, at specificity cut-off thresholds of 50% and 65%, respectively. The model was well calibrated with a calibration-in-the-large of 0.009 and calibration slope of 0.952. The Brier score was 0.025 indicting good overall predictive performance. (Fig. 2). Decision curve analysis showed comparable net benefit between the development and validation cohorts, indicating consistent model performance (Appendix 2.)

### Improving undertriage

The prediction model could potentially improve undertriage rates in the external validation cohort from 29.6% to 8.7% (∆ difference, 20.9%) and to 14.8% (∆ difference, 14.8%) at an overtriage rate of 50% and 35%, respectively. Threshold probabilities with corresponding under- and overtriage rates are shown in Appendix 3.

## Discussion

In this prospective, multicenter cohort study, a novel pre-hospital triage prediction model was developed and externally validated to identify patients with severe injury using a GBDT model. The prediction model demonstrates the potential to further reduce undertriage rates in the derivation and validation regions and was well calibrated. Incorporating the novel prediction model in the TTApp might therefore further improve pre-hospital patient allocation and could increase the TTApp’s applicability, as the prediction model was specifically designed to make predictions in urgent situations.

So far, identifying patients in need of specialized trauma care is predominantly based on field triage protocols, which are flowchart like structures unable to distinguish the interdependent relationships of signs and symptoms of severe injury, resulting in limited sensitivity in identifying patients in need of specialized trauma care [8]. Several studies have developed prediction models to identify patients in need of specialized trauma care, using both traditional statistical approaches and, more recently, machine learning techniques. However, these models often predict specific outcomes (e.g., hemorrhage risk or need for hemorrhage control) or are derived from selected populations (e.g., helicopter-transported patients or those directly transported to higher-level trauma centers), limiting their generalizability to general trauma triage and identification of patients requiring transport to a higher-level trauma center [17–22]. We previously developed a pre-hospital prediction model to select severely injured patients (ISS ≥ 16) at the scene of injury, which was able to potentially reduce undertriage rates to 11.2%, at an overtriage rate of 50% in the development cohort [9]. When integrated in the TTApp and implemented during a pilot study in approximately 25% of the Dutch trauma regions, undertriage rates decreased from 31.8% to 26.8%, while overtriage rates did not increase (20.4%) [10]. The previous model was, however, developed based on a relatively small cohort (4950 patients of which 435 patients had an ISS ≥ 16), and was based on a logistic regression model which required complete data availability to provide predictions. The current prediction model was developed using a substantially larger cohort of 51,001 trauma patients (of whom 1238 patients had an ISS ≥ 16) and, when externally validated, yielded undertriage rates of 8.7% and 14.8% at overtriage rates of 50% and 35%, respectively, lower than those reported for our previous TTApp prediction model and other published pre-hospital triage prediction models. Advantageously, the novel model was specifically designed to make predictions in urgent situation without the necessity of complete data availability, as GBDT models deal with missing data by default. The use of these Machine Learning models allows the TTApp to make real-time predictions and continuously update its recommendation when new data becomes available, as predictors such as dispatch priority and vital signs can be automatically gathered from the digital EMS records. This reduces the need for manual input and prevents deriving EMS professionals from their routine of care and comprehensively increases the TTApp’s applicability. Due to both the improved performance and pre-hospital applicability, incorporating the novel prediction model in the TTApp is expected to further improve pre-hospital patient allocation and reduce undertriage. The prediction model allows EMS regions to regulate patient allocation to region specific acceptable overtriage rates to prevent extensive shifts in patient allocation when the TTApp is integrated in the Dutch pre-hospital practice.

### Strengths and limitations

This study has several strengths. First, the prospective data collection and inclusion of all adult trauma patients transported by two ambulance services to both higher- and lower-level trauma centers in three trauma regions is a strength of this study, as it allowed us to develop a prediction model within a large generalizable trauma population and externally validate this model in an adjacent EMS region. Second, the use of a GBDT model is a great strength of this study, as these models generally provide highly accurate predictions and can handle missing data by default unlike logistic regression models. The use of these Machine Learning models is, due to these features, especially ideal in pre-hospital triage settings as it (1) decreases the need for manual input, (2) allows the model to perform when certain parameters (e.g., vital signs) are not measurable or filled in, and (3) is subsequently able to provide real-time predictions.

This study also has some limitations. First, the pre-hospital suspicion of serious injuries was determined by trained research assistants who read all pre-hospital free texts and is in future use of the TTApp subjective to the EMS professionals’ primary assessments at the scene of injury. Extensive instructions on the practical use of the TTApp will be conducted prior to the nationwide implementation to ensure consistency and adequate use of the app. Second, while the use of a GBDT model improves the performance and applicability of the novel prediction model, limitations related to missing data remain. In future clinical settings, both the extend and patterns of missing data may differ from those observed in this study, which could impact the model’s performance and generalizability [23]. Although the model can generate predictions with incomplete input, extensive missingness may be associated with reduced predictive precision. The impact of varying degrees of data completeness on model performance will therefore be examined during implementation. Third, our prediction model was designed to identify severe injury, defined as an ISS ≥ 16. This outcome is widely used to guide the need for transfer to a higher-level trauma center and is recommended by the ACSCOT for evaluating pre-hospital triage. However, prior research suggests that ISS may not fully align with a patient’s actual need for early critical resources or interventions, potentially limiting its effectiveness as sole criterion for triage decision-making [24]. Future iterations of the TTApp may therefore incorporate alternative outcomes, such as early critical resource use [25].

The utilization of the TTApp facilitates the start of precision-medicine in pre-hospital trauma triage, and is intended to be implemented in the Netherlands in the coming years [3]. Future research should evaluate the accuracy of pre-hospital patient allocation following the TTApp’s implementation and continue the improvement strategy as new data becomes available.

## Conclusion

A novel prediction model was developed as a first iteration of updating the TTApp’s performance and demonstrate to surpass the previous model and potentially improve undertriage rates towards 10% at acceptable overtriage rates. The prediction model was specifically designed to make predictions in urgent situations without the necessity of complete data input. Incorporating this model in the TTApp might further improve pre-hospital patient allocation and initiates a continuous improvement strategy.

## Supplementary Information

Below is the link to the electronic supplementary material.

Supplementary Material 1

Supplementary Material 2

Supplementary Material 3

Supplementary Material 4

## Data Availability

The data that supports the findings of the current study is not publicly available due to its sensitive nature but is available upon a reasonable request that needs to be approved by the participating Emergency Medical Services and trauma regions, provided that appropriate ethical approval is sought. R-scripts are available upon request.

## References

[1] MacKenzie EJ, Rivara FP, Jurkovich GJ, et al. A national evaluation of the effect of trauma–center care on mortality. N Engl J Med. 2006;354:366–78. 10.1056/NEJMsa052049. 16436768 10.1056/NEJMsa052049

[2] Newgard CD, Staudenmayer K, Hsia RY, et al. The cost of overtriage: more than one–third of low–risk injured patients were taken to major trauma centers. Health Aff (Millwood). 2013;32(9):1591–9. 10.1377/hlthaff.2012.1142. 24019364 10.1377/hlthaff.2012.1142 PMC4044817

[3] Zorginstituut Nederland. Advies bevordering implementatie multitraumanorm - verder omdat het beter is. Published March 28, 2023. https://www.zorginstituutnederland.nl/publicaties/adviezen/2023/03/28/advies-multitraumanorm. Accessed 13 Jun 2023.

[4] American College of Surgeons Committee on Trauma. Resources for the optimal care of the injured patient. Available from: https://www.facs.org/quality-programs/trauma/quality/verification-review-and-consultation-program/standards/. Accessed 13 Jun 2023.

[5] van Rein EAJ, van der Sluijs R, Houwert RM, et al. Effectiveness of prehospital trauma triage systems in selecting severely injured patients: is comparative analysis possible? Am J Emerg Med. 2018;36(6):1060–9. 10.1016/j.ajem.2017.11.016. 29395772 10.1016/j.ajem.2018.01.055

[6] Ambulancezorg Nederland. Landelijk protocol ambulancezorg. https://www.ambulancezorg.nl/themas/kwaliteit-van-zorg/protocollen-en-richtlijnen/landelijk-protocol-ambulancezorg. Accessed 13 Jun 2023.

[7] Newgard CD, Fischer PE, Gestring M, et al. National guideline for the field triage of injured patients: Recommendations of the National Expert Panel on Field Triage, 2021. J Trauma Acute Care Surg. 2022;93(2):e49–60. 10.1097/TA.0000000000003627. 35475939 10.1097/TA.0000000000003627 PMC9323557

[8] Voskens FJ, van Rein EAJ, van der Sluijs R, et al. Accuracy of prehospital triage in selecting severely injured trauma patients. JAMA Surg. 2018;153(4):322–7. 10.1001/jamasurg.2017.4472. 29094144 10.1001/jamasurg.2017.4472 PMC5933379

[9] van Rein EAJ, van der Sluijs R, Voskens FJ, et al. Development and validation of a prediction model for prehospital triage of trauma patients. JAMA Surg. 2019;154(5):421–9. 10.1001/jamasurg.2018.4752. 30725101 10.1001/jamasurg.2018.4752 PMC6537785

[10] Lokerman RD, van Rein EAJ, Waalwijk JF, et al. Accuracy of prehospital triage of adult patients with traumatic injuries following implementation of a trauma triage intervention. JAMA Netw Open. 2023;6(4):e236805. 10.1001/jamanetworkopen.2023.6805. 37014639 10.1001/jamanetworkopen.2023.6805 PMC10074221

[11] Ambulancezorg Nederland. Tabellenboek 2018. Available from: https://www.ambulancezorg.nl/sectorkompas/sectorkompas-2018. Accessed 13 Jun 2023.

[12] van der Sluijs R, Lokerman RD, Waalwijk JF, et al. Accuracy of pre–hospital trauma triage and field triage decision rules in children (P2–T2 study): an observational study. Lancet Child Adolesc Health. 2020;4(4):290–8. 10.1016/S2352-4642(19)30431-6. 32014121 10.1016/S2352-4642(19)30431-6

[13] Gennarelli TA, Wodzin E, Association for the Advancement of Automotive Medicine. Abbreviated Injury Scale 2005: Update 2008. Barrington, IL: Association for the Advancement of Automotive Medicine; 2008.

[14] Chen T, Guestrin C. XGBoost: a scalable tree boosting system. arXiv. Published March 3, 2016. Available from: https://arxiv.org/abs/1603.02754. Accessed 15 Apr 2025.

[15] R Core Team. R: A Language and Environment for Statistical Computing. Vienna, Austria: R Foundation for Statistical Computing; 2021.

[16] Vickers AJ, Elkin EB. Decision curve analysis: a novel method for evaluating prediction models. Med Decis Mak. 2006;26(6):565–74. 10.1177/0272989X06295361. 10.1177/0272989X06295361 PMC2577036 17099194

[17] Morris R, Karam BS, Zolfaghari EJ, et al. Need for emergent intervention within 6 hours: a novel prediction model for hospital trauma triage. Prehosp Emerg Care. 2022;26(4):556–65. 10.1080/10903127.2021.1984360. 34313534 10.1080/10903127.2021.1958961

[18] Dinh MM, Bein KJ, Oliver M, et al. Refining the trauma triage algorithm at an Australian major trauma centre: derivation and internal validation of a triage risk score. Eur J Trauma Emerg Surg. 2014;40(1):67–74. 10.1007/s00068-013-0292-4. 26815779 10.1007/s00068-013-0315-1

[19] Weidman AC, Malakouti S, Salcido DD, et al. A Machine Learning Trauma Triage Model for Critical Care Transport. JAMA Netw Open. 2025;8(6):e259639. 10.1001/jamanetworkopen.2025.9639. 40489113 10.1001/jamanetworkopen.2025.9639 PMC12150194

[20] Chen Q, Qin Y, Jin Z, et al. Enhancing Performance of the National Field Triage Guidelines Using Machine Learning: Development of a Prehospital Triage Model to Predict Severe Trauma. J Med Internet Res. 2024;26:e58740. 10.2196/58740. 39348683 10.2196/58740 PMC11474124

[21] Frock A, Robbins JT, Vital-Lopez FG, et al. A case study of AI-enabled software as a medical device cleared by the FDA for assessing hemorrhage risk index (APPRAISE-HRI) after trauma. NEJM AI. 2025;2(11):e2401170. 10.1056/aics2401170. 10.1056/aics2401170 PMC12981239 41836285

[22] Gauss T, James A, Colas C, et al. Comparison of machine learning and human prediction to identify trauma patients in need of hemorrhage control resuscitation (ShockMatrix study): a prospective observational study. Lancet Reg Health Eur. 2025;55:101340. 10.1016/j.lanepe.2025.101340. 40584589 10.1016/j.lanepe.2025.101340 PMC12205608

[23] Groenwold RHH. Informative missingness in electronic health record systems: the curse of knowing. Diagn Progn Res. 2020;4:8. 10.1186/s41512-020-00082-z. 32699824 10.1186/s41512-020-00077-0 PMC7371469

[24] Baxt WG, Upenieks V. The lack of full correlation between the Injury Severity Score and the resource needs of injured patients. Ann Emerg Med. 1990;19(12):1396–400. 10.1016/S0196-0644(05)82606-X. 2240752 10.1016/s0196-0644(05)82606-x

[25] Lerner EB, Willenbring BD, Pirrallo RG, et al. A consensus–based criterion standard for trauma center need. J Trauma Acute Care Surg. 2014;76(4):1157–63. 10.1097/TA.0000000000000189. 24662885 10.1097/TA.0000000000000189

