# Supplementary material for: Development and external validation of a novel prediction model for the TraumaTriage App
Source: Eur J Trauma Emerg Surg. 2026 Apr 17;52(1):135. doi: 10.1007/s00068-026-03175-8 (PMC13090252; doi:10.1007/s00068-026-03175-8)
Supplement: Supplementary file 1 — Supplementary Material 1 [file 68_2026_3175_MOESM1_ESM.docx]

**Appendix 1.** Model development

A Gradient Boosting Decision Tree model was developed to predict severe injury (ISS ≥ 16). This ensemble learning technique combines multiple decision trees to iteratively refine predictions, where each decision tree is sequentially trained with the residuals of the previous tree to adjust for the errors in the previously estimated model and improve the accuracy of the subsequent model.^16^ Predictor selection was based on prior literature and clinical relevance, with inclusion restricted to variables readily available in the pre-hospital setting to ensure feasibility of real-time implementation. The model included age, vital signs (systolic blood pressure, oxygen saturation, and Glasgow Coma Scale), high-energy mechanism, prehospital suspicion of severe injury per body region (head/neck, thorax, abdomen, pelvis, extremities, or multiple regions), and ambulance dispatch priority. The model was developed using data from EMS regions Brabant, while external validation was performed on data from a geographically distinct EMS region (Utrecht), ensuring separation between development and validation cohorts. Missing data were handled inherently by the gradient boosting algorithm, which allows for surrogate splits and does not require prior imputation, thereby reflecting real-world prehospital data conditions. Model training was performed using 5-fold cross-validation to optimize performance and reduce the risk of overfitting. The optimal number of trees was selected based on the minimum cross-validated root mean squared error (RMSE). Hyperparameters were selected based on cross-validation performance to balance model complexity and generalizability. In the final model, hyperparameters were sat at a maximum tree depth of 6, learning rate of 0.3, minimal child weight of 1, subsample of 1, and the optimal number of trees was 22. Model performance was evaluated in terms of discrimination (c-statistic), calibration (calibration-in-the-large and calibration slope), and overall performance (Brier score).

Information about the hyperparameters is available at: <https://xgboost.readthedocs.io/en/latest/parameter.html>
