# Supplementary material for: Development and external validation of a novel prediction model for the TraumaTriage App
Source: Eur J Trauma Emerg Surg. 2026 Apr 17;52(1):135. doi: 10.1007/s00068-026-03175-8 (PMC13090252; doi:10.1007/s00068-026-03175-8)
Supplement: Supplementary file 2 — Supplementary Material 2 [file 68_2026_3175_MOESM2_ESM.docx]

| **Appendix 2.** Threshold probabilities and discriminative ability of the prediction model in the development and external validation region | | | | | | | |
| --- | --- | --- | --- | --- | --- | --- | --- |
| **Threshold probability** | Sensitivity, % | Specificity, % | Undertriage, % | Overtriage, % | Positive predictive value | Negative predictive value |  |
|  |  |  |  |  |  |  |  |
| **Development region** |  |  |  |  |  |  |  |
| 0.005 | 99.1 | 24.6 | 0.9 | 75.4 | 0.032 | 0.999 |  |
| 0.0075 | 97.9 | 35.1 | 2.1 | 64.9 | 0.036 | 0.999 |  |
| 0.01 | 95.6 | 47.3 | 4.4 | 52.7 | 0.043 | 0.998 |  |
| 0.0125 | 92.2 | 57.7 | 7.8 | 42.3 | 0.051 | 0.997 |  |
| 0.015 | 89.4 | 65.1 | 10.6 | 34.9 | 0.060 | 0.996 |  |
| 0.0175 | 87.6 | 68.6 | 12.4 | 31.4 | 0.065 | 0.996 |  |
| 0.02 | 85.6 | 71.8 | 14.4 | 28.2 | 0.070 | 0.995 |  |
| 0.03 | 72.8 | 85.4 | 27.2 | 14.6 | 0.110 | 0.992 |  |
| 0.04 | 65.6 | 90.3 | 34.4 | 9.7 | 0.144 | 0.991 |  |
| 0.05 | 62.0 | 92.4 | 38.0 | 7.6 | 0.168 | 0.990 |  |
| 0.10 | 47.0 | 97.0 | 53.0 | 3.0 | 0.284 | 0.987 |  |
|  |  |  |  |  |  |  |  |
| **External validation region** |  |  |  |  |  |  |  |
| 0.005 | 97.0 | 31.3 | 3.0 | 68.7 | 0.043 | 0.997 |  |
| 0.0075 | 93.5 | 42.9 | 6.5 | 57.1 | 0.050 | 0.995 |  |
| 0.01 | 90.7 | 51.5 | 9.3 | 48.5 | 0.056 | 0.994 |  |
| 0.0125 | 87.8 | 59.6 | 12.2 | 40.4 | 0.065 | 0.994 |  |
| 0.015 | 84.3 | 66.8 | 15.7 | 33.2 | 0.075 | 0.992 |  |
| 0.02 | 80.2 | 71.2 | 19.8 | 28.8 | 0.082 | 0.991 |  |
| 0.03 | 78.6 | 75.4 | 21.4 | 24.6 | 0.093 | 0.991 |  |
| 0.04 | 69.7 | 84.1 | 30.3 | 15.9 | 0.122 | 0.989 |  |
| 0.05 | 63.7 | 88.3 | 36.3 | 11.7 | 0.148 | 0.987 |  |
| 0.10 | 58.7 | 90.7 | 41.3 | 9.3 | 0.168 | 0.986 |  |
|  |  |  |  |  |  |  |  |
|  |  |  |  |  |  |  |  |
| The prediction model was developed in the Brabant region and externally validated in the Utrecht region. | | | | | | | |

**Appendix 3.** Threshold probabilities and discriminative ability
