# Supplementary material for: Development and external validation of a novel prediction model for the TraumaTriage App
Source: Eur J Trauma Emerg Surg. 2026 Apr 17;52(1):135. doi: 10.1007/s00068-026-03175-8 (PMC13090252; doi:10.1007/s00068-026-03175-8)
Supplement: Supplementary file 3 — Supplementary Material 3 [file 68_2026_3175_MOESM3_ESM.docx]

**Appendix 2.** Decision curve analysis
