# Supplementary figures and images for: Development and external validation of a novel prediction model for the TraumaTriage App
Source: Eur J Trauma Emerg Surg. 2026 Apr 17;52(1):135. doi: 10.1007/s00068-026-03175-8 (PMC13090252; doi:10.1007/s00068-026-03175-8)

**Appendix 4.** Field triage criteria of the Dutch National Protocol of Ambulance Services


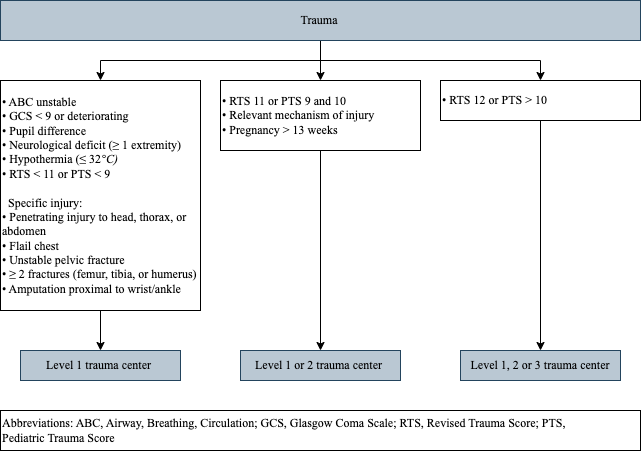

Supplement: Supplementary file 4 — Supplementary Material 4 [file 68_2026_3175_MOESM4_ESM.docx]
